# Perioperative nutrition-oriented nursing for older women undergoing cancer surgery: frailty screening, enhanced recovery, and survivorship care

**DOI:** 10.3389/fpubh.2026.1949710

**Published:** 2026-09-17

**Authors:** Yun Feng, Feng Yuan, Bingqing Kong

**Affiliations:** 1 Department of Anesthesiology and Operating Room, Zhejiang Hospital, Hangzhou, Zhejiang, China; 2 Department of Thoracic Surgery, Zhejiang Hospital, Hangzhou, Zhejiang, China

**Keywords:** enhanced recovery after surgery, frailty, older women with cancer, oncology nursing, perioperative nutrition, prehabilitation, sarcopenia, survivorship

## Abstract

Older women undergoing cancer surgery commonly enter the perioperative period with overlapping frailty, malnutrition, sarcopenia, obesity, multimorbidity, and treatment-related symptoms. These vulnerabilities reduce physiologic reserve and amplify the catabolic response to surgery, increasing the risk of complications, functional decline, delayed adjuvant treatment, and persistent nutritional impairment. Nutrition-oriented perioperative nursing provides a practical framework for translating frailty assessment and cancer nutrition guidance into coordinated care before surgery, during enhanced recovery, and after discharge. This Mini Review examines the frailty-malnutrition-surgical stress axis in older women with breast, gynecologic, thoracic, and other cancers requiring surgery. We propose an integrated two-step approach in which brief frailty and nutrition screening triggers multidimensional assessment, dietitian referral, symptom management, protein-energy support, and multimodal pre-habilitation. Within enhanced recovery pathways, nurses can protect nutritional intake by limiting unnecessary fasting, supporting individualized carbohydrate use, preventing postoperative nausea and vomiting, promoting early protein-containing oral feeding, and coordinating early mobilization. Continued reassessment after discharge is required because muscle loss, appetite disturbance, and treatment-related nutritional problems may persist into adjuvant therapy and survivorship. Current evidence supports integrated screening and multimodal care, but women-specific trials in geriatric oncology remain limited. Future studies should evaluate nurse-led implementation using functional, nutritional, treatment-continuity, and patient-reported outcomes rather than length of stay alone.

## 1 Introduction

Cancer surgery is increasingly performed in older women, including those with breast, endometrial, ovarian, cervical, vulvar, lung, and gastrointestinal malignancies. Chronologic age alone does not determine surgical resilience. Frailty, malnutrition, sarcopenia, multimorbidity, obesity, polypharmacy, cognitive vulnerability, and social support collectively shape the ability to tolerate surgery and recover function (1–7). In women, menopause-related changes in body composition and bone health may coexist with obesity or sarcopenic obesity, making low muscle reserve difficult to recognize from body mass index alone.

Nutrition is a modifiable component of this vulnerability. Cancer-related inflammation, reduced intake, gastrointestinal symptoms, depression, pain, and preoperative treatment can accelerate muscle loss. Malnutrition defined by the Global Leadership Initiative on Malnutrition (GLIM) criteria is associated with poorer survival and more postoperative complications in cancer populations, while frailty independently predicts mortality, complications, prolonged hospitalization, and non-home discharge after cancer surgery (8–13). In gynecologic oncology, malnutrition and sarcopenia are common and are associated with adverse postoperative or survival outcomes (14–19).

A tailored focus on older women is warranted because postmenopausal endocrine and body-composition changes can reduce muscle and bone reserve while increasing the likelihood of sarcopenic obesity that is not apparent from body mass index alone. Women-specific cancer surgery and treatment sequelae may coexist with widowhood, living alone, caregiving obligations, and restricted access to food preparation, transportation, or rehabilitation. These intersecting biological and social vulnerabilities are not consistently represented in mixed-sex perioperative studies and support a sex and gender-responsive nursing pathway (10, 18, 20).

Perioperative nurses are positioned at the interface of assessment, surgical preparation, anesthesia, symptom control, early feeding, mobilization, discharge education, and continuity of care. A nutrition-oriented nursing model does not replace dietitians, geriatricians, anesthesiologists, surgeons, or rehabilitation professionals. Its role is to ensure that nutritional and functional vulnerability is recognized early, translated into an actionable pathway, and monitored across transitions. This Mini Review integrates frailty screening, nutritional optimization, enhanced recovery after surgery (ERAS), and survivorship-oriented follow-up for older women undergoing cancer surgery.

## 2 Why older women are nutritionally vulnerable before cancer surgery

Older women with cancer frequently present with mixed nutritional phenotypes. Some have visible weight loss and low body mass, whereas others have obesity with depleted muscle quantity or quality. Prospective and meta-analytic evidence in gynecologic cancer demonstrates that sarcopenia may be present despite normal or elevated body mass index and is associated with worse survival (15–17). In women aged 80 years or older with gynecologic malignancy, frailty and comorbidity have stronger relationships with treatment tolerance and survival than age alone (18).

The preoperative period can intensify these risks. Diagnostic procedures, neoadjuvant therapy, pain, nausea, early satiety, constipation, anxiety, and restrictions imposed by patients or caregivers may reduce energy and protein intake. Inflammatory signaling and insulin resistance promote anabolic resistance, while inactivity accelerates loss of muscle strength. Albumin is influenced by inflammation and should not be used as a stand-alone nutritional diagnosis, but low albumin and weight loss remain clinically useful warning signals when interpreted with intake, phenotype, and disease burden. A systematic two-step process—rapid screening followed by multidimensional assessment—is therefore preferable to isolated laboratory testing (7–10).

Nutrition vulnerability also has a gendered and social dimension. Older women may live alone, depend on caregivers for shopping and food preparation, or prioritize family responsibilities over their own intake. Financial constraints and transportation barriers may limit access to oral nutritional supplements, dietitians, or pre-habilitation. Nursing assessment should therefore include food access, oral health, swallowing, gastrointestinal symptoms, cultural food practices, health literacy, and the feasibility of implementing recommendations at home.

## 3 Integrated frailty and nutrition screening as a nursing pathway

Screening should occur when surgery is first planned, not on the evening before the procedure. A brief frailty tool such as the Clinical Frailty Scale or FRAIL scale can be paired with a validated nutrition screen such as NRS-2002, the Malnutrition Screening Tool, or the abridged Patient-Generated Subjective Global Assessment. Frailty identified by simple scales is associated with postoperative complications, delirium, and mortality, but no single instrument captures all relevant domains (11–13). Positive screening should trigger comprehensive geriatric and nutritional assessment rather than function as a reason to deny surgery.

The second step should characterize potentially reversible deficits. Relevant components include recent weight change, usual intake, symptom burden, muscle strength, mobility, cognition, mood, comorbidity, medication burden, social support, and the planned surgical stress. GLIM diagnosis requires at least one phenotypic and one etiologic criterion (8). Handgrip strength, chair-stand performance, gait speed, or calf circumference can complement the assessment. When preoperative computed tomography is already available, opportunistic assessment of skeletal muscle may identify sarcopenia or myosteatosis, but imaging should not delay care.

Nurses can operationalize risk stratification using the practical pathway summarized in Table 1. Low risk indicates negative frailty and nutrition screens with stable intake and function; moderate risk indicates a positive screen or a potentially reversible decline; and high risk indicates established malnutrition, frailty, marked intake reduction, sarcopenia, or major functional dependence. Each level should trigger a predefined referral and follow-up plan rather than serve as a reason to deny surgery.

**Table 1:** Practical frailty-nutrition risk stratification and nursing response.

Risk level	Screening tools and positive findings	Referral or escalation	Priority nursing actions
Low	CFS 1–3 or FRAIL 0; NRS-2002 < 3 and MST < 2; stable weight, intake, mobility, and cognition	Routine perioperative pathway; repeat screening if clinical status changes	Standard ERAS education; reconcile fasting and feeding instructions; monitor intake, symptoms, and mobility
Moderate	CFS 4 or FRAIL 1–2; NRS-2002 ≥ 3 or MST ≥ 2; recent weight, intake, strength, or mobility decline	Dietitian and physiotherapy referral; consider geriatric or medical review	Symptom-directed counseling; protein-energy support; home-based pre-habilitation; scheduled preoperative reassessment
High	CFS ≥ 5 or FRAIL ≥3; GLIM-defined malnutrition, marked intake reduction, sarcopenia, or major functional/cognitive dependence	Multidisciplinary review involving nutrition, geriatrics, anesthesia, surgery, and rehabilitation; consider optimization before surgery when oncologically safe	Individualized nutrition and pre-habilitation plan; early postoperative feeding and mobilization; documented discharge plan and early post-discharge follow-up

Thresholds are pragmatic examples based on commonly used tools (7, 8, 10–13). Positive screens should prompt multidimensional assessment and do not independently define malnutrition or preclude surgery. CFS, Clinical Frailty Scale; ERAS, enhanced recovery after surgery; FRAIL, Fatigue, Resistance, Ambulation, Illnesses, and Loss of weight; GLIM, Global Leadership Initiative on Malnutrition; MST, Malnutrition Screening Tool; NRS-2002, Nutritional Risk Screening 2002.

## 4 The frailty-malnutrition-surgical stress axis

Frailty and malnutrition interact with the surgical stress response through a common metabolic pathway. Aging, cancer inflammation, endocrine change, low intake, and inactivity reduce muscle reserve and anabolic responsiveness. Surgical trauma then activates sympathetic and hypothalamic–pituitary–adrenal pathways, releasing catecholamines and cortisol and increasing inflammatory mediators such as IL-6, TNF-alpha, and C-reactive protein. Hyperglycemia, pain, fasting, nausea, and immobility further increase insulin resistance and suppress intake (21).

In a patient with adequate reserve, this response is usually reversible. In a frail older woman, the same stress may cross a functional threshold. Proteolytic pathways and dysregulated autophagy accelerate muscle breakdown, while mitochondrial dysfunction limits energy production. Reduced protein availability impairs wound healing and immune competence; prolonged fasting and opioid-related gastrointestinal dysfunction delay feeding; and postoperative inactivity reinforces muscle loss. Immediate postoperative muscle loss has been associated with inadequate protein and energy intake (22).

This axis provides a mechanistic rationale for coordinated nursing interventions (Figure 1). Prehabilitation increases reserve before surgery; ERAS reduces avoidable stress during the perioperative period; early protein-containing nutrition and mobilization limit postoperative catabolism; and longitudinal monitoring prevents short-term deterioration from becoming chronic disability. The intervention target is therefore not a single nutrient but the interaction among intake, muscle, inflammation, symptoms, and function.

**Figure 1:**
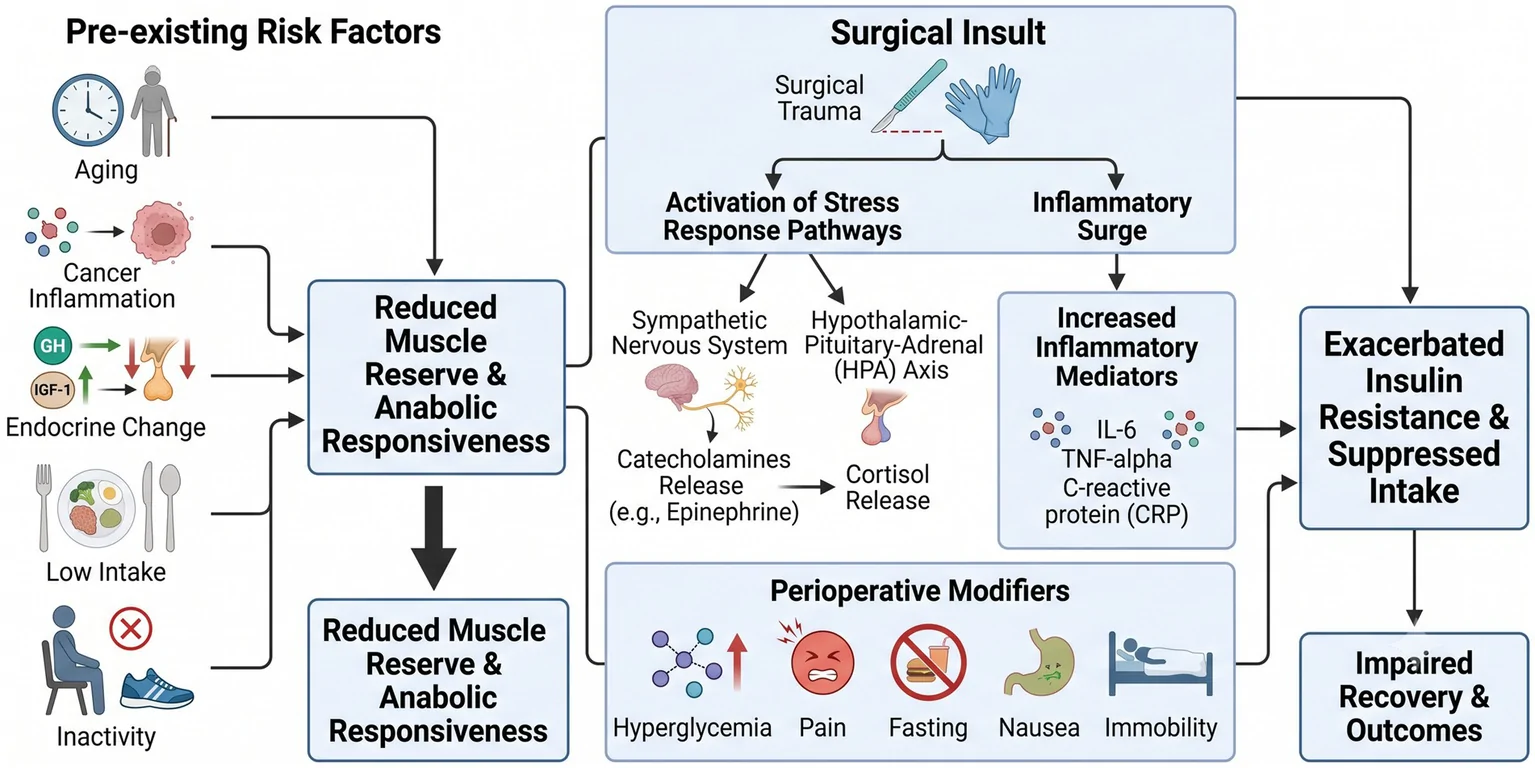
Frailty-malnutrition-surgical stress axis and interruption points for perioperative nutrition-oriented nursing in older women undergoing cancer surgery. Aging, cancer-related inflammation, endocrine change, reduced intake, comorbidity, and inactivity reduce muscle and physiologic reserve. Surgical trauma amplifies cortisol and catecholamine release, inflammatory signaling, insulin resistance, fasting, nausea, and immobility, promoting muscle proteolysis, autophagy imbalance, mitochondrial dysfunction, and impaired immune, wound, and gastrointestinal recovery. Nutrition-oriented nursing interrupts this pathway through integrated frailty and nutrition screening, protein-energy support, multimodal prehabilitation, reduction of unnecessary fasting, symptom control, early protein-containing oral nutrition, mobilization, and longitudinal reassessment.

## 5 Nutrition-oriented nursing within enhanced recovery

Preoperative phase. Education should explain why nutrition and activity are part of surgical treatment. Nurses should reconcile conflicting fasting instructions, identify nausea, constipation, dysphagia, pain, or anxiety that limits intake, and verify that referred women can access dietetic care. Current pre-habilitation evidence suggests that exercise and nutritional support are important components, although certainty varies and frail cancer populations remain underrepresented (23–26). A recent multimodal program in women with gynecologic cancer improved physical fitness and reduced the proportion at moderate or high risk of malnutrition before surgery (27). For women with inadequate intake, oral nutritional supplements or food-based protein-energy enrichment may be used under dietetic guidance.

Immediate perioperative phase. The operating-room and anesthesia nursing team can protect recovery by implementing ERAS elements consistently: avoiding unnecessary prolonged fasting, confirming individualized use of preoperative carbohydrate drinks when appropriate, maintaining normothermia and euvolemia, supporting glycemic control, and enabling opioid-sparing analgesia. Prevention of postoperative nausea and vomiting is a nutrition intervention because uncontrolled symptoms delay drinking and eating. ERAS programs in gynecologic surgery reduce length of stay, readmission, and ileus without evidence of harm, and can be implemented in women with obesity or major procedures when supported by experienced teams (28–30).

Postoperative phase. Oral intake should resume as early as clinically feasible rather than waiting routinely for bowel sounds or flatus. Immunonutrition may reduce infectious complications in selected upper gastrointestinal cancer surgery, but evidence is not sufficiently women-specific to support indiscriminate use (31). Meta-analyses support early oral feeding after gastrointestinal surgery, and protein-containing diets may be more clinically relevant than non-nutritive clear fluids (32, 33). Early enteral feeding after pelvic exenteration has also been shown to be feasible and beneficial for gastrointestinal recovery (34). Nurses should assess swallowing, nausea, pain, ileus, diarrhea, early satiety, and food acceptability; document actual intake rather than diet orders alone; and escalate persistent deficits promptly.

Early mobilization must be coordinated with nutrition. Feeding without muscle loading may not restore function, while exercise without adequate intake may deepen energy deficit. For older women with cancer who are malnourished, sarcopenic, or unable to meet needs through usual intake, approximately 1.2 g/kg/day may be considered a contextual lower target rather than a universal prescription (35). The selected target and body-weight metric should be individualized by the dietitian or clinical nutrition team according to renal function, cancer type, disease burden, obesity or edema, treatment plan, and physical activity. Immediate postoperative muscle loss has also been associated with inadequate protein and energy intake, reinforcing the need to document actual intake and coordinate nutrition with muscle loading (22).

Breast-cancer perioperative nursing literature emphasizes structured education, symptom control, functional support, and continuity across the surgical pathway (36). Patient-experience evidence also indicates that inconsistent information, limited communication, and poor acceptability of postoperative diets can reduce engagement with ERAS and nutrition pathways; nursing messages and diet progression should therefore be consistent, individualized, and reinforced across transitions (37, 38).

## 6 From discharge to survivorship care

Discharge is not the end of perioperative nutrition care. Older women may experience appetite loss, altered taste, constipation, diarrhea, fatigue, wound problems, or practical difficulty preparing food after returning home. Some cancer operations create durable changes in digestion or absorption, and postoperative muscle loss can persist well beyond hospital discharge (39). The transition is particularly vulnerable when adjuvant chemotherapy or radiotherapy begins before nutritional recovery is complete.

A survivorship-oriented pathway should specify who reassesses weight, intake, muscle function, and frailty; when escalation occurs; and how information moves between surgical oncology, primary care, community nursing, rehabilitation, and dietetics. Individualized dietary counseling after cancer surgery can improve energy and protein goal attainment, reduce weight loss, and improve quality-of-life domains (40). Evidence from women aged 80 years or older with endometrial cancer further supports incorporating comorbidity and functional status into follow-up planning rather than relying on chronological age alone (41). Telephone or digital follow-up may support symptom monitoring, but should not replace face-to-face assessment for women with cognitive, sensory, or digital-access barriers.

Nursing outcomes should extend beyond length of stay. Relevant measures include nutritional risk, handgrip strength, chair-stand performance, mobility, and treatment completion, time to adjuvant therapy, readmission, patient confidence, caregiver burden, and quality of life. Older female cancer survivors with stress, depression, comorbidity, and low positive affect may have greater frailty, underscoring the need to integrate psychosocial support with nutrition and function (20). Perioperative nursing research should therefore treat survivorship as a continuation of recovery rather than a separate phase.

## 7 Implementation priorities and evidence gaps

The evidence base has three important limitations. First, many perioperative nutrition and pre-habilitation trials combine cancer sites and include relatively few older women. Findings from colorectal or upper gastrointestinal surgery cannot be transferred uncritically to breast and gynecologic oncology. Second, screening tools and sarcopenia definitions vary, limiting comparison and implementation. Third, intervention studies often measure short-term complications and length of stay but not muscle preservation, independence, adjuvant-treatment continuity, or long-term quality of life.

Future trials should recruit risk-enriched groups, including women aged 70 years or older, those with ovarian or endometrial cancer, obesity with low muscle mass, prior neoadjuvant treatment, and limited social support. Interventions should report the exact nutritional dose, exercise prescription, symptom-management strategy, and nursing contact time. Pragmatic studies should compare a clearly defined nurse-coordinated pathway with usual care and include implementation outcomes such as screening completion, referral uptake, time to intervention, fidelity, equity, and cost.

Clinical implementation can begin before definitive women-specific trials are complete. Institutions can standardize a two-step frailty-nutrition screen, create automatic referral thresholds, align fasting and feeding instructions across disciplines, monitor actual postoperative intake, and schedule reassessment after discharge. These actions are consistent with current geriatric oncology, cancer nutrition, and surgical nutrition guidance (3–5, 7). Experience in obese gynecologic oncology populations also suggests that ERAS pathways can be implemented when metabolic, anesthetic, nutritional, and mobility needs are individualized (42).

## 8 Conclusion

Older women undergoing cancer surgery face a convergent risk of frailty, malnutrition, sarcopenia, surgical stress, and incomplete recovery. Nutrition-oriented perioperative nursing converts this risk profile into an actionable continuum: integrated screening, pre-habilitation, reduced perioperative stress, early protein-containing feeding, mobilization, symptom control, and survivorship follow-up. The approach should be individualized, multidisciplinary, and evaluated by preservation of function and treatment continuity as well as conventional surgical outcomes. Women-specific geriatric oncology trials are needed, but routine nursing practice can already ensure that nutritional vulnerability is identified early and not lost at transitions of care.
